# Supplementary material for: Comprehensive Annotation and Functional Exploration of MicroRNAs in Lettuce
Source: Front Plant Sci. 2021 Dec 24;12:781836. doi: 10.3389/fpls.2021.781836 (PMC8739914; doi:10.3389/fpls.2021.781836)
Supplement: Supplementary file 2 [file Data_Sheet_8.ZIP › GSM2230748.plot/Lsa-miRN86_Lsat_1_v5_gn_8_41821.1_455_TPlot.pdf]

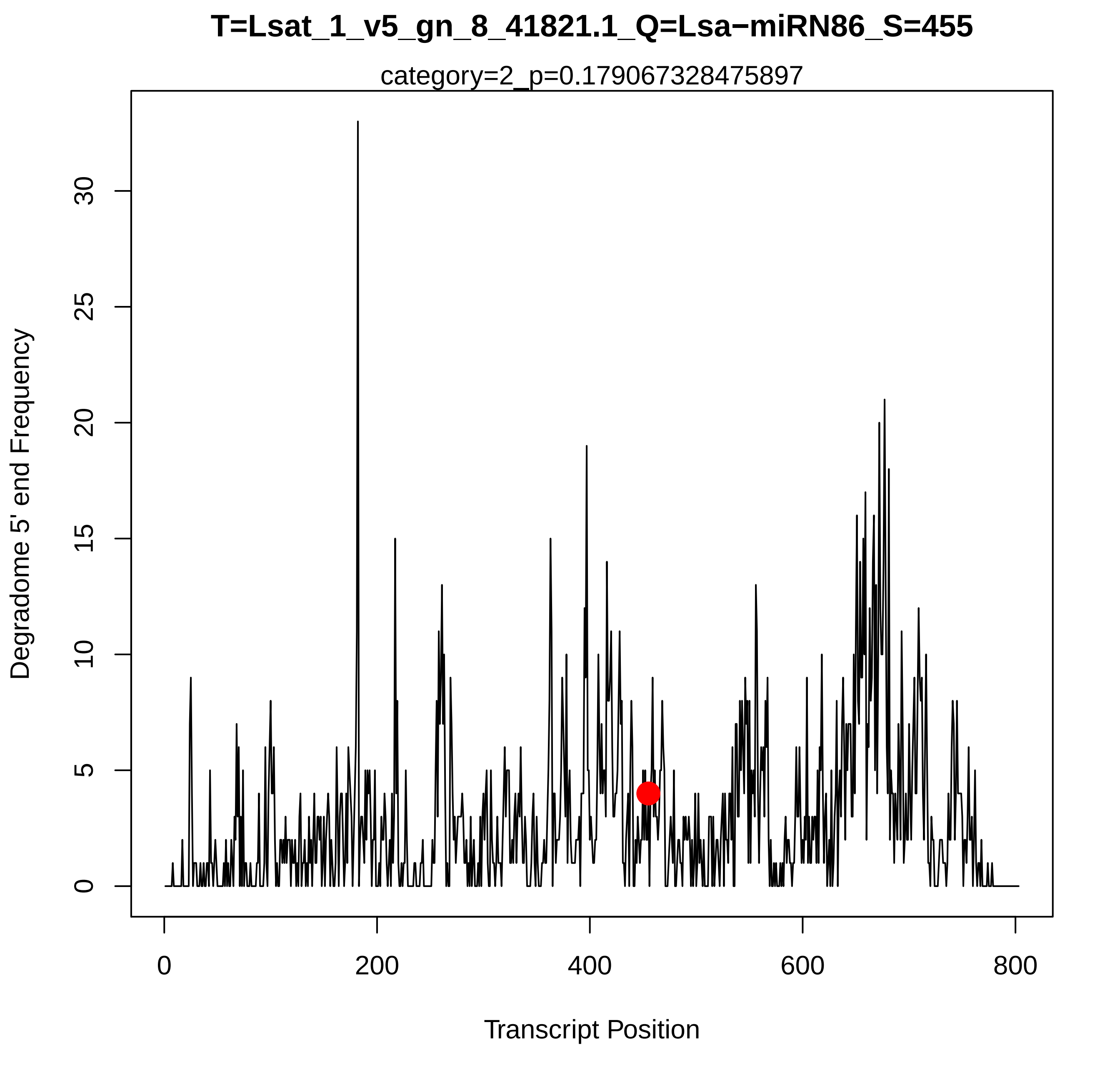

T=Lsat_1_v5_gn_8_41821.1_Q=Lsa−miRN86_S=455
category=2_p=0.179067328475897
Degradome 5' end Frequency
Transcript Position
0
5
10
15
20
25
30
0
200
400
600
800
